# Supplementary material for: Interpretable deep learning of myelin histopathology in age-related cognitive impairment
Source: Acta Neuropathol Commun. 2022 Sep 21;10:131. doi: 10.1186/s40478-022-01425-5 (PMC9490907; doi:10.1186/s40478-022-01425-5)
Supplement: Supplementary file 1 — Additional file 1: Figure S1 Annotation procedure of blue hue ranges in Luxol fast blue, hematoxylin, and eosin-stained tiles. Representative tiles show the annotation method used for positive pixel counting in the Luxol fast blue, hematoxylin, and eosin (LH&E) stained histology tiles. For the annotation heatmap, the darker blue pixel range is highlighted as red while the lighter blue pixel range is highlighted as light blue. Figure S2 Correlation of slide-level probability estimates of cognitive impairment in matched brain donors between the two brain regions. Scatter plots showing the probability estimates of cognitive impairment by the top-performing models in the same brain donors between WSIs in the hippocampus and frontal cortex data sets. The blue line shows predictions from a linear model and grey error envelopes show 95% confidence intervals for the linear model. Figure S3 Scatterplot matrix of deep histopathology features with clinicopathologic features in the frontal cortex. Correlation analysis of deep histopathology results and clinicopathologic features: age, Braak score, ARTAG positivity in the hippocampus (coded as 0 = not present and 1 = present), cognitive label (coded as 0 = not cognitively impaired and 1 = cognitively impaired), probability of cognitive impairment as predicted by the top-performing model trained on the frontal cortex data, and median LFB staining intensity (pixel counts) in the top attention tiles in the frontal cortex data set. Upper right: rank correlation values and associated p-values (* = p < 0.05, ** = p < 0.01, *** = p < 0.001). Diagonal: histograms of variables. Lower left: Scatterplots with linear model trend lines for the variable pairs (red lines) and 95% confidence intervals (blue envelopes). This plot was made using the R package GGally (v. 2.1.2). CI = Cognitive impairment; ARTAG = Aging-related tau astrogliopathy; LFB = Luxol Fast Blue. [file 40478_2022_1425_MOESM1_ESM.docx]

**Supplementary Figures**


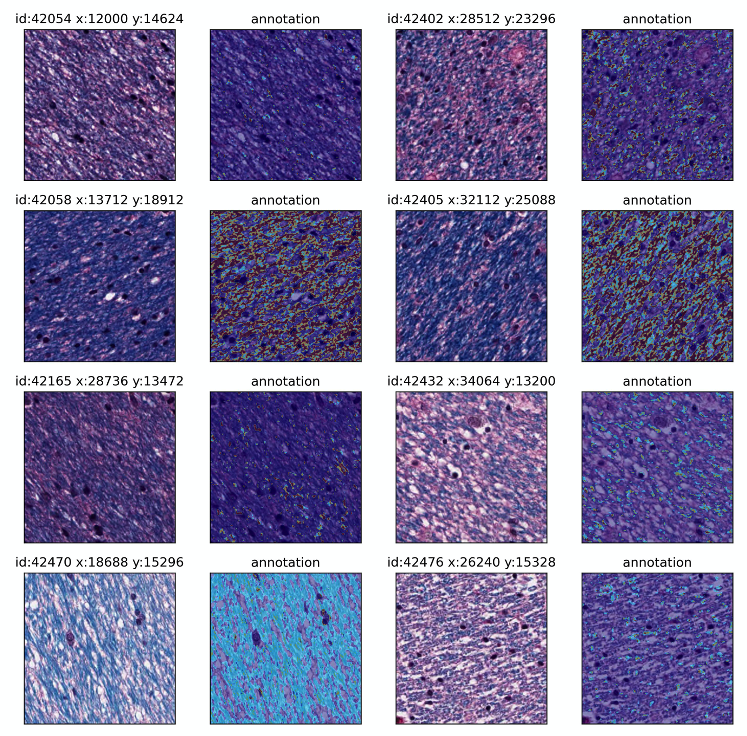


**Supplementary Figure 1.** Annotation procedure of blue hue ranges in Luxol fast blue, hematoxylin, and eosin-stained tiles.

Representative tiles show the annotation method used for positive pixel counting in the Luxol fast blue, hematoxylin, and eosin (LH&E) stained histology tiles. For the annotation heatmap, the darker blue pixel range is highlighted as red while the lighter blue pixel range is highlighted as light blue.

**Supplementary Figure 2.** Correlation of slide-level probability estimates of cognitive impairment in matched brain donors between the two brain regions.

Scatter plots showing the probability estimates of cognitive impairment by the top-performing models in the same brain donors between WSIs in the hippocampus and frontal cortex data sets. The blue line shows predictions from a linear model and grey error envelopes show 95% confidence intervals for the linear model.

**Supplementary Figure 3.** Scatterplot matrix of deep histopathology features with clinicopathologic features in the frontal cortex.

Correlation analysis of deep histopathology results and clinicopathologic features: age, Braak score, ARTAG positivity in the hippocampus (coded as 0 = not present and 1 = present), cognitive label (coded as 0 = not cognitively impaired and 1 = cognitively impaired), probability of cognitive impairment as predicted by the top-performing model trained on the frontal cortex data, and median LFB staining intensity (pixel counts) in the top attention tiles in the frontal cortex data set. Upper right: rank correlation values and associated p-values (* = p < 0.05, ** = p < 0.01, *** = p < 0.001). Diagonal: histograms of variables. Lower left: Scatterplots with linear model trend lines for the variable pairs (red lines) and 95% confidence intervals (blue envelopes). This plot was made using the R package GGally (v. 2.1.2). CI = Cognitive impairment; ARTAG = Aging-related tau astrogliopathy; LFB = Luxol Fast Blue.
